# Modulation of nutrient composition of black soldier fly (*Hermetia illucens*) larvae by feeding seaweed-enriched media

**DOI:** 10.1371/journal.pone.0183188

**Published:** 2017-08-24

**Authors:** Nina S. Liland, Irene Biancarosa, Pedro Araujo, Daan Biemans, Christian G. Bruckner, Rune Waagbø, Bente E. Torstensen, Erik-Jan Lock

**Affiliations:** 1 National Institute of Nutrition and Seafood Research, Bergen, Norway; 2 Protix Biosystems BV, Industriestraat 3, Dongen, The Netherlands; 3 Norwegian Institute of Bioeconomy Research (NIBIO), Torggården, Bodø, Norway; Biocenter, Universität Würzburg, GERMANY

## Abstract

Black soldier fly (*Hermetia illucens*) larvae are a promising source of protein and lipid for animal feeds. The nutritional composition of the BSF larvae depend partly on the composition of the feeding medium. The BSF lipid profile in part mimics the feeding media lipid profile, and micronutrients, like minerals and vitamins, can readily accumulate in black soldier fly larvae. However, investigative studies on bioconversion and accumulation of nutrients from media to black soldier fly larvae are scarce. Here we show that inclusion of the brown algae *Ascophyllum nodosum* in the substrate for black soldier fly larvae can introduce valuable nutrients, commonly associated with the marine environment, into the larvae. The omega-3 fatty acid eicosapentaenoic acid (20:5n-3), iodine and vitamin E concentrations increased in the larvae when more seaweed was included in the diet. When the feeding media consisted of more than 50% seaweed, the larvae experienced poorer growth, lower nutrient retention and lower lipid levels, compared to a pure plant based feeding medium. Our results confirm the plasticity of the nutritional make-up of black soldier fly larvae, allowing it to accumulate both lipid- and water-soluble compounds. A broader understanding of the effect of the composition of the feeding media on the larvae composition can help to tailor black soldier fly larvae into a nutrient profile more suited for specific feed or food purposes.

## Introduction

The larva of black soldier fly (BSF, *Hermetia illucens*) is a scavenger, commonly used to accelerate composting of organic material. BSF larvae can efficiently utilize organic resources, like fruit, vegetable and meat waste [1]. The BSF larvae can, depending on the feeding medium, contain high concentrations of lipids (>30% of dry weight, dw) and protein (around 40% of dw) [2,3]. Recently, interest in using BSF larvae as a raw material for animal feed has increased, mainly due to its potential as a sustainable source of high-quality protein [4,5].

Fish oil is currently the most important resource for the health-promoting marine omega-3 polyunsaturated fatty acids (PUFA) in fish feed. Total landings of global fisheries remains stable at best [6] and the global production of fish oil is unlikely to increase in the years to come. Finding other resources for the marine PUFA is imperative to meet future growing demand from both human and animal feed markets of long-chain omega-3. Typical marine fatty acids are 20:5n-3 eicosapentaenoic acid (EPA) and 22:6n-3 docosahexaenoic acid (DHA). A global demand for sources of nutrients associated with the marine environment, such as the EPA, DHA and iodine, requires better use of the traditional marine resources as well as finding new sources of these nutrients. Resources not currently being used directly as food can potentially be refined and used as feed ingredients for livestock and farmed fish. One such resource is wild and cultured seaweed, commonly used in parts of Asia for food. There is a great potential in both harvest and culture of seaweeds also in Europe [7]. Seaweed is, however, not optimal as a feed ingredient for many animals due to its high content of water, salt and complex carbohydrates [8].

In the current study, feeding media using varying inclusions of brown algae (*Ascophyllum nodosum*) were used in a regression design growth trial with BSF larvae. *A*. *nodosum* was chosen due to its high availability, with large natural stocks available throughout the year from subtropical to arctic regions within the Northern Atlantic [9]. Up to 100% of a plant-based feeding medium, optimized for protein growth in industrial production of BSF, was gradually replaced with ground seaweed. Seaweeds are usually low in lipids (0.9–3.7% of dry weight) and do not contain DHA, but in some species EPA make up as much as 34% of the fatty acids [10]. Seaweeds also contain iodine, sterols, essential amino acids and vitamin E [11]. BSF larvae can be enriched in marine omega-3 PUFAs (final concentration in larvae at ~2% EPA + DHA of total fatty acids) when fed on fish offal [12]. The insect larvae can therefore be carriers of such essential nutrients from sources not directly suitable for human- or animal nutrition, an ability that can be used to tailor the composition of the insect larvae towards desired nutrient profiles to be used as feed ingredients. The aim of this study was to investigate the conversion of carbohydrate-rich seaweed into lipid and protein by BSF larvae, and to assess the possible transfer of desirable marine nutrients from the seaweed to the insect larvae.

## Materials and methods

### Experimental set-up

The control feeding medium (BA0) for the insect larvae consisted of a plant-based material consisting of processed wheat (particle size: 50–2000 μm), used as a reference material for black soldier fly larvae growth by Protix Biosystems BV (The Netherlands). The pure seaweed feeding medium (BA100) consisted of 100% brown algae (*A*. *nodosum*) prepared as described below. The trial followed a regression design, where the basic feeding medium (BA0) was gradually replaced with the seaweed feeding medium in steps of 10%. The design of the experiment as well as replicate crates for each treatment are described in Table 1. A proposed regression design at eleven concentration levels and different replication per level was compared against the ideal six concentration levels in triplicate as described elsewhere [13,14]. The comparison of the two experimental settings (S1 Table) indicated that the uncertainty of the proposed design is consistently lower than the ideal regression design over the entire concentration range. In addition, BSF larvae were fed on BA0 (four crates, n = 4) and transferred to BA100 24 hours before harvesting (24H), to detect how quickly the insect larvae could take up nutrients from the feeding medium.

**Table 1:** Description of the 11 different feeding media prepared for the black soldier fly larvae growth trial, replacing from 10% up to 100% of a plant-based feeding medium with brown algae.

	BA0	BA10	BA20	BA30	BA40	BA50	BA60	BA70	BA80	BA90	BA100
**BA in medium**	0%	10%	20%	30%	40%	50%	60%	70%	80%	90%	100%
**Replicates**^a^	4	1	1	1	1	2	1	1	1	1	4

BA: brown algae

^a^ Number of crates where the given feeding medium was used.

#### Seaweed collection and processing

The seaweed biomass consisted of 100% *A*. *nodosum* (L.) Le Jolis, harvested in April 2015 near Bodø, Norway. Whole thalli were collected in plastic bags and kept between 0°C and 5°C during transport. At the laboratory, the algae were rinsed in cold freshwater to remove adhering foreign material and frozen at -20°C, followed by grinding to a particle size of 0.5–2 mm and freezing at -30°C. At the facility of Protix Biosystems BV (Dongen, The Netherlands), the ground seaweed biomass was thawed and mixed with the plant-based control medium according to the experimental design, a sample of each feeding medium was frozen for analysis.

#### Rearing of BSF larvae

The experiment was carried out at the facility of Protix Biosystems BV with 8 days old BSF larvae (from the Protix BSF colony). Eighteen experimental units (crates of size 40x30x15cm) were added their respective feeding media and ~15 000 BSF larvae (individual weight ~13.7 mg). Feeding medium was added daily until the control group (BA0) reached harvest size (individual larvae weight ~100 mg, total feeding period was 8 days). The larvae were kept in complete darkness during the experiment, only while feed was added light was present. Feeding media was added depending on how much uneaten media was left in the crates at feeding and according to the size of the larvae. Total feed added per crate was recorded daily. At the end of the trial, total weight of larvae and waste material were recorded per crate. The larvae were grown at 30°C with 65% relative humidity.

At the end of the eight-day growth period the larvae were separated from the feeding media by manual sieving and immediately preserved by freezing on dry ice. Samples from the waste material were collected and immediately frozen on dry ice. Total waste material was weighed and recorded. Frozen samples were ground to a powder by using a blender (Knife Mill Grindomix GM 100, Retsch, Haan, Germany). Dry ice was added in the blending process to prevent thawing of samples. Aliquots of the samples were freeze-dried (as described under chemical analysis). All samples were stored at -30°C until analysed.

The feeding media, harvested larvae and compost left after removal of larvae (waste) were lyophilized for the content of dry matter by first freezing 24 h at -20°C in vacuum (0.2–0.01 mBar) and then leaving in vacuum at 25°C until constant weight. Total lipid (ethyl acetate extraction) and ash content (550°C, overnight) was measured gravimetrically in wet, ground samples of larvae.

### Chemical analyses

Analysis of total amino acids (excluding cysteine and tryptophan) of feeding media, larvae and waste material after harvesting the larvae was carried out by ultra performance liquid chromatography (UPLC, Waters Acquity UPLC system) coupled with a UV detector [15,16]. Wet, powdered samples containing 30–40 mg of protein were hydrolysed in 6M HCl at 110°C for 22 hours. Prior to hydrolysis, 3.125 mM Norvaline (Sigma-Aldrich, St. Louis, MO, USA) was added as internal standard, and 0.1M Dithiothreitol (DTT, Sigma-Aldrich) as an antioxidant agent to protect methionine from degradation during acid hydrolysis. For a further protective aid, sample tubes were topped up with nitrogen gas. During acid hydrolysis, cysteine and tryptophan are destroyed and are therefore not reported in the results. After hydrolysis, samples were cooled to room temperature and centrifuged in a vacuum centrifuge until complete dryness was reached. After centrifugation, the residue was diluted in MilliQ-Plus water and filtered through a syringe-driven filter. Prior to the instrumental analysis, a derivatisation agent (AccQ.Tag^™^, Waters, Milford, MA, USA) was added to each sample. Finally, amino acids were separated by UPLC (column: Aquity UPLC BEH C18 1.7 μM, Waters, flowrate 0.7 mL min^-1^) and results integrated by Empower 3 (Waters).

Total nitrogen was analysed on freeze-dried, ground samples using a CHNS elemental analyser (Vario Macro Cube, Elementar Analysensysteme GmbH, Langenselbold, Germany) and quantified according to Dumas (1831). The instrument was calibrated with EDTA (Leco Corporation, Saint Joseph, MI, USA). Sulfanilamide (Alfa Aesar GmbH & Co, Karlsruhe, Germany) and a standard meat reference material (SMRD 2000, LGC Standards, Teddington, UK) were used as control samples.

Non-Protein Nitrogen Compounds of the insect larvae were analysed on wet, powdered samples with a Biochrom 20 Plus amino acid analyser (Biochrom, Cambourne, United Kingdom) according to manufacturer’s instructions. The column used was a Biochrom Physiological column (200 mm) and the results integrated by Empower 3 (Waters).

Fatty acid composition was determined for feeding media, larvae and the waste material left after the larvae were harvested as described by Torstensen *et al*. [17] based on [18]. Briefly, lipids were extracted from wet, grinded samples by homogenisation in chloroform:methanol (2:1, v:v) and analysed using gas chromatography coupled with a flame ionisation detector. The following instrumentation was used: Autosystem XL (Perkin Elmer, Waltham, MA, USA) with pre-column Silica 0.53 mm i.d. (Imperial Eastman Tubing, Baltimore, MD, USA) and a CP-sil-88^™^ column, 50 m*0.32 mm i.d. Helium was used as carrier gas at 1.5 ml min^-1^ and hydrogen as a detector gas at 45 ml min^-1^. The peaks were identified with the software Chromeleon^®^ version 6.8 (Dionex, Sunnyvale, CA, USA) and individual methyl esters were identified by comparison to known standards and on the basis of published values [19]. Quantification of fatty acids was done by using 19:0 methyl ester as an internal standard.

Vitamin E species in the insect larvae were analysed by high-performance liquid chromatography (HPLC) according to [20] as described by [21]. 0.1–1 g of wet, powdered sample was added 0.01 g of ascorbic acid, 0.01 g pyrogallol, 4 mL ethanol, 0.5 mL saturated EDTA and 0.5 mL 20% KOH (w:v in distilled water). Samples were mixed well and boiled 20 minutes at 100°C. Room-tempered samples were added 1 ml distilled water and tocopherols extracted three times with 2 mL hexane:ethylacetate (80:20, v:v:). Samples were diluted in hexane prior to analysis on an HPLC system coupled with a fluorescence detector (Ultimate 3000 series, Dionex, Sunnyvale, CA, USA). Column dimensions were 3 μm 150 x 2 mm (Pinnacle DB Silica) and separation performed at a flow rate of 0.3 mL min^-1^) The tocopherol and tocotrienol species were detected and quantified by external standards after running an external standard mixture with the sample set.

Mineral concentrations in freeze-dried, grinded material were analysed by inductively coupled plasma–mass spectrometry (ICP-MS) after wet digestion in a microwave oven, as described by Julshamn *et al*. [22] with some modifications. Shortly, the samples were digested in 69% nitric acid (2 mL) and 30% hydrogen peroxide (0.5 mL) using a microwave digestion system (UltraWAVE, Milestone, Sorisole, Italy). The solutions were diluted to 25 mL with deionized water (MilliQ, Merck Millipore, Billerica, MA, USA). Mineral concentrations in the samples were quantified by ICP-MS (iCapQ ICPMS, ThermoFisher Scientific, Waltham, MA, USA) equipped with an autosampler (FAST SC-4Q DX, Elemental Scientific, Omaha, NE, USA). Data were collected and processed using the Qtegra ICPMS Software (ThermoFisher Scientific). Iodine concentrations were determined in freeze-dried, grinded material. Weighed sample (0.2–0.5 g) was added 5 mL water and 1 mL tetramethylammonium hydroxide (TMAH, 1% in water) and kept at 90°C for three hours. The samples were then diluted with 25 mL water, centrifuged five minutes at 3500 rpm and the supernatant filtered through a 0.45μm syringe filter. Iodine concentrations were determined by ICP-MS (Agilent 7500, Agilent, Santa Clara, CA, USA) coupled with an autosampler (ASX-500 series, Cetac, Omaha, NE, USA) as described by [22]. Data were collected and processed using the Agilent Mass hunter Workstation software.

Calculations of nutrient productive value (NPV) for individual larvae crates were done as shown in formula below. NPV > 1 indicates a net production of a nutrient by the larvae.

$$\text{NPV}=\frac{\text{g nutrient in larvae}}{\text{g nutrient in medium}-\text{g nutrient in waste}}$$

The NPV was calculated for protein (PPV), amino acids (AAPV) and selected fatty acids (FAPV).

Retention of nutrients was calculated as follows:

$$\text{\% nutrient retention}=\frac{\text{g nutrient in larvae}}{\text{g nutrient added}}\;\;\times \;100$$

Best-fit regression lines were found using the linear model (lm) function in the free software environment R [23]. The data were analysed for homogeneity in variance using a Levene’s test and for normality using a Shapiro Wilk’s test. A Welch two sample t-test was used to detect differences between the BA0 control group and the 24H group. Data are presented as mean ± SD and a significance level of 95% was used. Figures were made using GraphPad Prism version 7.01 for Windows (GraphPad Software, La Jolla, CA, USA).

## Results

### Composition of feeding media

Since the crude protein estimation using N content x 6.25 has shown to overestimate the actual protein content in plant materials and seaweeds [15,24], the true protein values (sum of anhydrous amino acids) are presented for the feeding media. In the feeding media, ~30–40% of the nitrogen was of non-protein origin, while it was 30–56% in the waste. The true protein content decreased from 10.8% of dry feeding media in the BA0 to 4.5% in the BA100 medium (Table 2). More seaweed in the feeding media also led to some smaller changes in amino acid composition (Table 2). Alanine, aspartic acid and glutamic acid increased as % of total amino acids when introducing seaweed to the feeding media, while histidine, leucine, valine, arginine, glycine, proline, serine and tyrosine decreased.

**Table 2:** Proximate-, amino acid- and fatty acid composition of feeding media used for the black soldier fly larvae growth trial.

	BA0	BA10	BA20	BA30	BA40	BA50	BA60	BA70	BA80	BA90	BA100
**Proximate composition, %**											
Dry matter	31.1	31.0	30.9	30.8	30.5	30.0	30.3	29.4	32.8	28.4	28.2
Protein^a^, dm	10.8	9.8	9.6	8.6	8.5	7.4	6.7	6.5	5.1	5.3	4.5
**Amino acid composition, % of total amino acids**											
**Essential amino acids**											
Histidine	2.7	2.6	2.6	2.5	2.5	2.3	2.2	2.2	1.8	1.5	1.1
Isoleucine	4.0	4.0	4.0	3.9	4.0	3.9	4.0	4.0	3.8	3.8	3.8
Leucine	7.7	7.6	7.5	7.5	7.4	7.3	7.2	7.2	6.9	6.8	6.7
Lysine	5.6	5.8	5.5	5.6	5.3	5.4	5.5	5.4	5.4	5.2	5.1
Methionine	1.7	1.6	1.7	1.6	1.7	1.7	1.7	1.7	1.7	1.8	1.9
Phenylalanine	4.6	4.4	4.5	4.3	4.7	4.4	4.4	4.6	4.0	4.2	4.2
Threonine	4.3	4.4	4.4	4.5	4.6	4.6	4.7	4.7	4.8	4.8	4.8
Valine	5.9	5.9	5.8	5.7	5.7	5.6	5.7	5.7	5.3	5.2	5.1
**Non-essential amino acids**											
Alanine	6.3	6.5	6.3	6.5	6.4	6.6	6.6	6.7	7.0	7.2	7.3
Arginine	6.0	5.7	5.8	5.5	5.5	5.2	5.4	5.4	4.8	4.5	4.4
Aspartic acid	8.7	9.2	9.1	9.7	9.7	10.3	11.0	11.1	11.9	12.8	13.6
Glutamic acid	19.1	20.2	20.3	20.8	20.6	21.5	20.9	20.9	23.6	24.9	25.9
Glycine	6.4	6.1	6.3	6.1	6.3	6.1	6.1	6.0	5.7	5.5	5.3
Proline	7.6	7.2	7.0	6.8	6.6	6.3	5.9	5.8	5.1	4.4	3.7
Serine	5.5	5.4	5.5	5.5	5.5	5.5	5.2	5.0	5.3	4.8	4.6
Tyrosine	3.9	3.4	3.7	3.4	3.6	3.2	3.6	3.5	2.9	2.7	2.5
**Fatty acid composition, area %**											
12:0	0.6	0.7	0.5	0.4	0.3	0.2	0.3	0.2	0.1	<LOQ	<LOQ
14:0	0.2	0.8	1.5	1.9	2.6	3.5	5.7	5.2	6.5	8.3	10.4
16:0	22.6	22.6	21.2	20.8	19.7	18.7	16.4	16.5	14.5	12.8	11.0
18:0	1.4	1.5	1.3	1.4	1.3	1.2	0.9	1.0	0.8	0.7	0.5
Total SFA	25.6	26.8	25.7	25.7	25.1	24.5	24.0	23.8	22.9	22.7	22.7
16:1n-7	<LOQ	<LOQ	<LOQ	<LOQ	<LOQ	<LOQ	0.9	0.9	1.1	1.5	1.8
18:1n-9	10.7	12.6	13.4	15.9	17.8	19.8	19.5	23.7	26.9	29.4	31.3
Total MUFA	12.8	14.7	15.5	18.3	20.1	22.3	21.9	26.2	29.6	32.2	34.4
18:2n-6 LA	55.9	52.5	49.8	44.8	40.9	37.0	33.4	29.1	22.2	15.7	8.2
18:3n-3 ALA	5.3	5.0	4.9	4.2	4.1	3.9	4.6	3.7	3.3	3.2	3.2
18:4n-3	<LOQ	0.1	0.3	0.4	0.6	0.9	1.5	1.3	1.6	2.0	2.6
20:2n-6	<LOQ	0.2	0.3	0.4	0.6	0.8	0.7	1.0	1.2	1.4	1.5
20:4n-6 ARA	<LOQ	1.0	1.5	2.4	3.4	4.7	6.2	6.9	8.6	10.5	12.4
20:5n-3 EPA	<LOQ	0.4	0.8	1.1	1.7	2.2	3.5	3.4	4.2	5.2	6.6
Total n-3	5.3	5.7	6.2	6.2	6.9	7.7	10.3	9.4	10.5	12.0	14.1
Total n-6	56.0	53.6	51.8	47.9	45.3	42.9	41.1	37.8	33.1	28.8	23.6
Total PUFA	61.3	59.2	58.0	54.1	52.3	50.7	51.5	47.2	43.7	40.8	37.8
n-3/n-6	0.09	0.10	0.12	0.13	0.15	0.18	0.25	0.25	0.32	0.42	0.60
Total FA (%), dm	4.81	4.76	4.22	3.26	3.26	3.25	3.95	2.79	2.12	2.36	1.97

^a^True protein (sum of anhydrous amino acids);

BA0 to BA100: feeding media with 0% to 100% brown algae (0% = pure plant based medium); dm: dry matter; LA: linoleic acid, ALA: alpha-linolenic acid, EPA: eicosapentaenoic acid, DHA: docosahexaenoic acid, SFA: saturated fatty acids, MUFA: monounsaturated fatty acids, PUFA: polyunsaturated fatty acids, FA: fatty acid. LOQ: limit of quantification (fatty acids: 0.01 g kg^-1^ (wet weight) or 0.1 area %).

The control feeding medium (BA0) contained 48.1 g total fatty acids per kg (dry weight) (Table 2). By increasing inclusion of seaweed up to 100% (BA100), the total fatty acid concentration was reduced to 19.7 g per kg (dry weight). The major fatty acids of the BA0 medium were (in decreasing percentages): 18:2n-6 (56% of total fatty acids) > 16:0 (23%) > 18:1n-9 (11%) (Table 2). In the BA100 medium, the major fatty acids were (in decreasing percentages): 18:1n-9 (31%) > 20:4n-6 (12%) > 16:0 (11%) > 14:0 (10%). Thus, by including seaweed in the feeding media, the percentages of 16:0 and 18:2n-6 were reduced, while the percentages of 14:0 and 18:1n-9 increased. In addition, by adding seaweed to the feeding media, polyunsaturated long chained fatty acids like 20:4n-6 and 20:5n-3 were introduced to the feeding media.

The feeding medium without seaweed (BA0) contained the lowest levels of minerals of all the feeding media used in the trial (Table 3). The concentration of most of the minerals analysed increased in the media with increasing seaweed inclusion, especially calcium, sodium and magnesium. Iodine was below the quantification limit in the BA0 medium, but reached concentrations of 700 mg kg^-1^ in the BA100 medium. The concentrations of phosphorus, manganese, and copper decreased with increasing seaweed inclusions, while zinc and selenium remained largely unchanged.

**Table 3:** Mineral- and sterol composition of feeding media used for the black soldier fly larvae growth trial (all results on dry matter basis).

	BA0	BA10	BA20	BA30	BA40	BA50	BA60	BA70	BA80	BA90	BA100
*Minerals*											
**Ca**, g kg^-1^	2.4	4.0	5.2	6.6	7.6	9.1	9.2	10	13	14	15
**K**, g kg^-1^	10.0	12	11	12	13	14	15	17	17	18	19
**Mg**, g kg^-1^	1.8	2.5	2.9	3.4	4.0	4.5	5.0	6.1	6.5	7.0	7.7
**Na**, g kg^-1^	4.8	6.9	10	13	16	19	22	27	29	33	36
**P**, g kg^-1^	4.6	4.5	4.2	3.8	3.4	3.1	2.7	2.3	1.9	1.4	1.1
**I**, mg kg^-1^	<LOQ	61	120	190	260	330	410	470	530	610	700
**Cu**, mg kg^-1^	6.3	6.2	6.0	5.7	5.5	5.3	5.0	4.7	4.4	4.1	4.1
**Fe**, mg kg^-1^	230	265	265	285	290	295	310	250	320	300	430
**Mn**, mg kg^-1^	48	45	42	39	35	32	28	25	21	17	16
**Se**, mg kg^-1^	0.07	0.07	0.08	0.08	0.08	0.09	0.09	0.08	0.1	0.1	0.1
**Zn**, mg kg^-1^	42	43	43	43	45	44	45	46	46	46	46
**Sterols**, mg kg^-1^											
**Cholesterol**	83.1	-	-	-	-	-	-	-	-	-	28.3
**Desmosterol**	<LOQ	-	-	-	-	-	-	-	-	-	<LOQ
**Fucosterol**	58.5	-	-	-	-	-	-	-	-	-	2165
**Stigmasterol**	150	-	-	-	-	-	-	-	-	-	<LOQ
**β-sitosterol**	782	-	-	-	-	-	-	-	-	-	16.2
**Total sterols**	1073	-	-	-	-	-	-	-	-	-	2210

BA0 to BA100: feeding media with 0% to 100% brown algae (0% = pure plant based medium); LOQ: limit of quantification (iodine: 2 mg kg^-1^; desmosterol: 11.3 mg kg^-1^; stigmasterol; 7.4 mg kg^-1^).

The BA100 feeding medium contained high concentrations of fucosterol, a sterol typical for many seaweeds, while the BA0 feeding medium only contained this sterol in low quantities (Table 3). There were low concentrations of cholesterol in both feeding media.

### Growth and survival of the larvae

**Fig 1:**
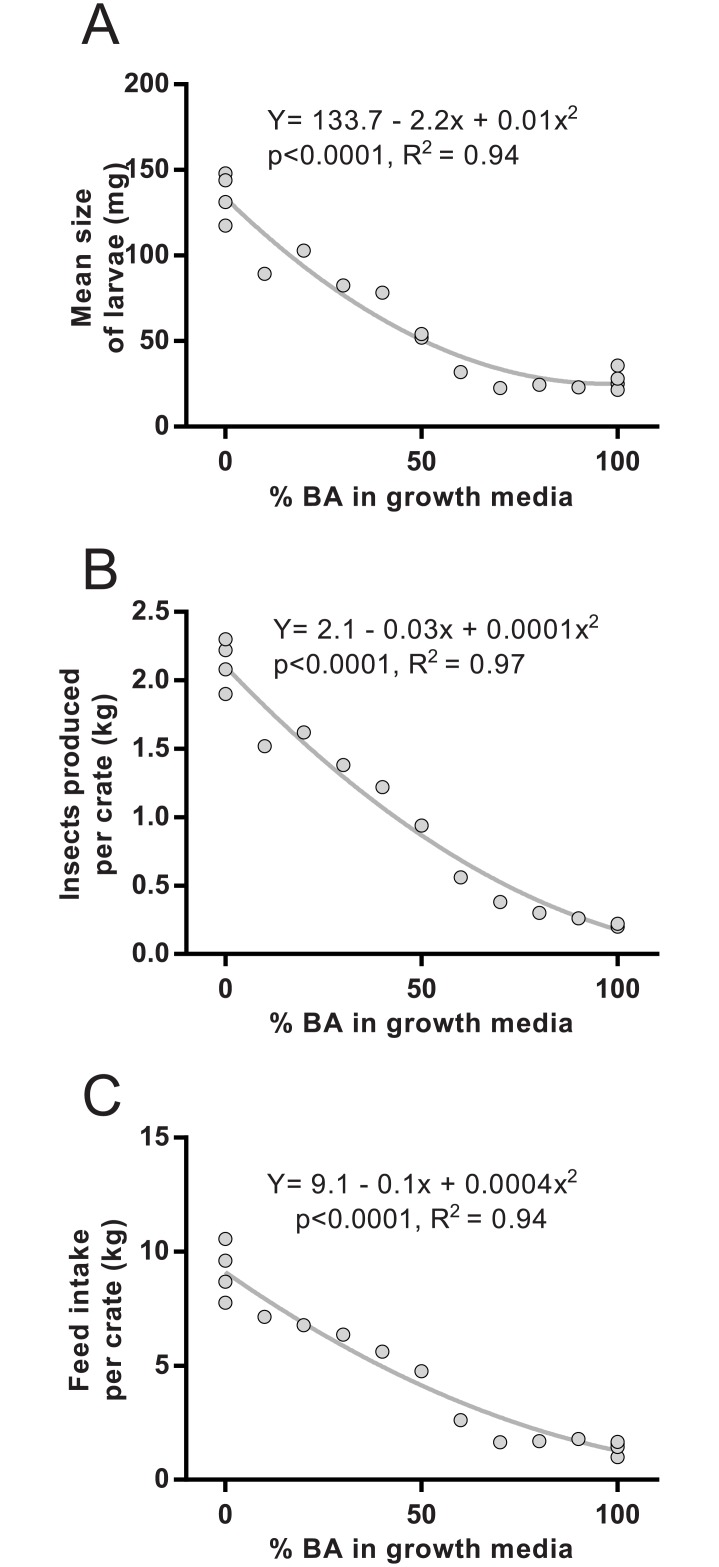
Growth of larvae. By increasing the inclusion of brown algae (BA) in the feeding medium, the black soldier fly larvae had reduced growth and feed intake. Figures are showing mean weight of individual larvae (wet weight) per crate by the end of the trial (A); kg larvae (wet weight) produced per crate during the trial (B) and total feeding media consumed/feed intake of the larvae (feeding medium added minus residue at end of trial) (C). BA: brown algae.

The larvae that were grown using seaweed in the feeding media were smaller than the larvae grown on the control medium, decreasing in size with increasing seaweed inclusions (individual weight of larvae in BA0: 135 ± 7 mg and in BA100: 28 ± 3 mg, Fig 1a). The crates with seaweed added also had reduced total mass of larvae per crate (yield) by the end of the trial (Fig 1b). The feed added per day gradually decreased in all the groups of larvae grown on more than 50% seaweed inclusion, leading to a large difference in total feed added in these groups (BA60 to BA100, Fig 1c). The conversion of feeding medium to larvae also decreased as more seaweed was included in the feeding media (BA0: 18.1 ± 0.5% of feeding medium converted into larvae, BA50: 16 ± 0.0%; BA100: 6.1 ± 0.1%). The larvae in the groups grown on higher inclusion of seaweed were also of a visibly darker colour and were more difficult to separate from the feeding media than the ones fed less seaweed. Of the ~15 000 larvae added to each crate, the majority (>95%) survived through the whole trial when grown on ≤70% inclusion. The survival dropped in the groups using more than 70% seaweed in the feeding media (BA80: 82%, BA90: 76%, BA100: 51 ± 5% survival).

### Chemical composition of larvae

The larvae grown on the control medium (BA0) consisted of ~37% dry matter, of which 29.1% was protein (true protein), 33% lipids and 5.1% ash (Table 4). Protein content of the insect larvae is presented both as true protein (sum of anhydrous amino acids) and crude protein. The crude protein content of the dried larvae was negatively affected at 50% seaweed inclusion, but not at 100% seaweed inclusion, however this effect was not significant for true protein. The ash content increased to 15.8% of dry matter in the larvae grown on BA100. Lipid content of the larvae was reduced to 7.7% by using 100% seaweed as feeding medium. By increasing the seaweed inclusion in the feeding media, the percentage of dry matter of the larvae decreased (~23% in BA100).

**Table 4:** Proximate composition (%, dry weight) and dry matter of black soldier fly larvae (% wet weight) grown on increasing inclusions of brown algae in feeding media.

	BA0^a^	BA10	BA20	BA30	BA40	BA50^b^	BA60	BA70	BA80	BA90	BA100^a^	P-value	Y =	R^2^
**Total lipid**	33.8 ± 1.6	n.a	n.a	n.a	n.a	22.2 ± 0.2	n.a	n.a	n.a	n.a	8.1 ± 0.9	<0.0001	34.0–0.3x	0.99
**True protein**^c^	29.1 ± 1.1	28.6	27.0	25.8	24.8	24.3± 0.7	27.7	28.6	28.9	26.9	29.2 ± 0.9	NS	-	-
**Crude protein**^d^	40.0 ± 0.9	37.9	35.9	35.3	33.5	33.7	37.4	42.3	41.0	39.3	41.3 ± 1.1	0.0001	40.0–0.3x + 0.003x^2^	0.91
**Ash**	5.1 ± 0.4	n.a	n.a	n.a	n.a	10.5	n.a	n.a	n.a	n.a	15.8 ± 0.7	<0.0001	1.9 + 0.04x - 0.0002x^2^	0.98
**Dry matter**	36.6 ± 0.5	35.6	35.2	32.6	32.8	30.7	27.7	23.3	24.0	23.3	23.1 ± 0.8	<0.0001	36.8–0.1x	0.99

^a^ n = 4,

^b^ mean value of two crates (n = 2),

^c^ sum of anhydrous amino acids,

^d^ nitrogen x 6.25;

BA0: insect larvae grown on plant-based control feeding medium; BA50 and BA100: insect larvae grown on feeding media where 50% and 100% of the control medium was replaced with ground brown algae. x = percent inclusion of brown algae in feeding media (0–100); n.a = not analysed

#### Amino acid composition of larvae

The most abundant amino acids in the larvae were aspartic acid and glutamic acid (Table 5). By increasing the percentage of seaweed in the media, the concentrations of lysine, methionine, phenylalanine, valine and aspartic acid of the larvae decreased (as % of crude protein). The concentration of glutamic acid in the larvae increased when more seaweed was included in the media. By using pure seaweed (BA100) as feeding medium for the last 24 hours of the trial, only the aspartic acid concentration changed, which decreased significantly from 9.4 in BA0 to 8.6 in the 24H group.

**Table 5:** Total amino acid composition (% of crude protein) of black soldier fly larvae grown on increasing inclusions of brown algae in feeding media.

	24H	BA0^a^	BA10	BA20	BA30	BA40	BA50^b^	BA60	BA70	BA80	BA90	BA100^a^	FM[25,26]	SP[25, 27]	BSF[28]	P value	Y =	R^2^
**Essential amino acids**																		
Histidine	2.6	2.8 ± 0.1	2.6	2.7	2.4	2.5	2.4	2.5	2.3	2.5	2.5	2.7 ± 0.1	2.1	1.3	3.3/2.7	NS	-	-
Isoleucine	3.9	3.9 ± 0.2	4.0	4.0	3.9	4.0	4.0	4.1	3.8	3.8	3.7	3.8 ± 0.1	5.1	4.0	4.2/4.7	NS	-	-
Leucine	6.2	6.4 ± 0.3	6.6	6.7	6.6	6.6	6.7	6.9	6.3	6.3	6.2	6.2 ± 0.1	6.5	6.1	6.6/7.1	NS	-	-
Lysine	5.9	6.2 ± 0.3	6.2	5.9	6.0	5.5	5.6	5.5	5.6	5.4	5.5	5.6 ± 0.3	10.1	3.7	5.9/6.0	<0.0001	6.28–0.02x + 0.0001x^2^	0.71
Methionine	1.6	1.7 ± 0.1	1.6	1.7	1.6	1.5	1.4	1.5	1.3	1.4	1.3	1.4 ± 0	3.1	1.4	1.6/1.7	<0.0001	1.66–0.003x	0.72
Phenylalanine	3.7	4 ± 0.2	3.9	4.3	3.8	3.7	3.4	3.6	3.0	3.2	3.0	3.2 ± 0.1	3.7	3.4	3.6/4.6	<0.0001	4.05–0.01x	0.75
Threonine	3.7	3.9 ± 0.1	4.0	4.1	4.0	3.9	4.0	4.1	3.7	3.9	3.9	3.9 ± 0.1	3.5	3.7	3.9/4.1	NS	-	-
Valine	5.7	5.8 ± 0.2	6.0	6.0	6.0	5.7	5.7	5.6	5.4	5.4	5.5	5.5 ± 0.2	4.5	4.4	5.7/6.4	0.001	5.88–0.003x	0.45
**Non-essential amino acids**																		
Alanine	6.3	6.2 ± 0.3	6.6	6.5	6.6	6.5	6.8	6.9	6.6	6.5	6.3	6.4 ± 0.2	6.1	3.7	7.8/6.9	NS	-	-
Arginine	4.5	4.6 ± 0.2	4.5	4.9	4.5	5.0	4.6	5.3	4.5	5.0	4.7	6.5 ± 3.2	5.2	5.1	4.8/6.1	NS	-	-
Aspartic acid	8.6*	9.4 ± 0.5	9.2	8.6	8.5	8.1	8.3	8.2	7.9	8.0	8.0	8.3 ± 0.4	9.3	11.5	8.2/8.5	<0.0001	9.43–0.04x + 0.03x^2^	0.74
Glutamic acid	10.3	10.3 ± 0.4	11.8	11.3	11.9	11.8	12.3	12.8	11.7	11.9	11.6	11.9 ± 0.5	13.1	20.7	11.8/8.7	<0.0001	10.42 + 0.058x - 0.0005x^2^	0.69
Glycine	4.8	4.6 ± 0.2	5.0	5.2	5.1	5.0	4.8	5.3	4.7	5.0	5.0	4.3 ± 2	6.6	3.7	5.6/5.2	NS	-	-
Proline	5.5	5.3 ± 0.2	5.8	5.8	5.8	5.9	5.9	6.1	5.4	5.5	5.0	5.2 ± 0.1	4.2	5.1	6.2/5.5	NS	-	-
Serine	4.0	4 ± 0.2	4.3	4.4	4.4	4.5	4.6	4.9	4.3	4.4	4.4	4.3 ± 0	4.4	5.3	4.3/3.9	NS	-	-
Tyrosine	5.3	5.7 ± 0.3	5.6	5.6	5.1	4.9	4.3	4.4	3.6	4.1	4.0	4.2 ± 0.1	2.9	3.2	5.1/7.1	<0.0001	5.84–0.04x + 0.0002x^2^	0.84

^a^ n = 4,

^b^ mean value of two crates (n = 2),

BA0 to BA100: insect larvae grown on media with 0% to 100% ground brown algae (0% = pure plant based medium); 24H: insect larvae fed media containing 100% brown algae only last 24 h before harvest; x = percent inclusion of brown algae in feeding media (0–100);

* Significantly different from the BA0 group (t-test);

FM: Fish meal; SP: Soy protein; BSF: Black soldier fly.

Due to low and highly varying concentrations of amino acids in the BA60-BA100 groups, which rendered unnaturally high or negative values, only the amino acid productive values (AAPVs) for the BA0-BA50 are presented (Table 6). The retention of single amino acids was, in general, slightly higher in the BA50 group than in the BA0 group, with significant increases in several of the AAPV. There was a tendency of the essential amino acids having higher AAPV than the non-essential amino acids. All essential amino acids, except for leucine and phenylalanine, had AAPV at ~1 or above for all crates, indicating a 100% retention or even a net production of some amino acids. The AAPV for taurine was the highest of all the amino acids, reaching values above 2, showing a clear net production of this amino acid by the insect larvae.

**Table 6:** Amino acid- and protein productive values (AAPV and PPV) of black soldier larvae grown on increasing inclusions of brown algae in feeding media.

	BA0^a^	BA10	BA20	BA30	BA40	BA50^b^	BA60-BA100	P value	Y =	R^2^
**Essential amino acids**										
Histidine	1.06 ± 0.04	1.36	1.19	1.32	0.98	1.35	NA*	NS	-	-
Isoleucine	0.90 ± 0.04	1.11	1.02	1.20	1.00	1.31	NA*	0.001	1.10 + 0.01x	0.73
Leucine	0.90 ± 0.03	1.11	1.02	1.20	1.00	1.31	NA*	0.002	0.92 + 0.007x	0.69
Lysine	1.38 ± 0.07	1.62	1.28	1.51	1.28	1.20	NA*	NS	-	-
Methionine	1.00 ± 0.04	1.21	1.12	1.25	0.94	1.06	NA*	NS	-	-
Phenylalanine	0.91 ± 0.04	1.17	1.07	1.13	0.76	1.05	NA*	NS	-	-
Threonine	1.09 ± 0.03	1.44	1.17	1.37	1.09	1.41	NA*	NS	-	-
Valine	1.12 ± 0.03	1.32	1.19	1.51	1.15	1.49	NA*	0.013	1.14 + 0.006x	0.50
**Non-essential amino acids**										
Alanine	1.44 ± 0.07	1.65	1.35	1.57	1.24	1.27	NA*	NS	-	-
Arginine	0.74 ± 0.03	0.97	0.91	1.01	0.90	1.16	NA*	0.0004	0.86 + 0.005x	0.78
Aspartic acid	1.52 ± 0.10	1.69	1.21	1.39	1.16	1.10	NA*	0.002	1.55–0.009x	0.67
Glutamic acid	0.59 ± 0.03	0.76	0.58	0.63	0.53	0.50	NA*	NS	-	-
Glycine	0.95 ± 0.05	1.62	1.19	1.45	0.95	1.16	NA*	NS	-	-
Proline	0.66 ± 0.03	1.03	0.91	1.01	0.86	1.01	NA*	0.009	0.72 + 0.006x	0.54
Serine	0.81 ± 0.03	1.16	0.93	1.08	0.92	1.12	NA*	0.024	0.86 + 0.005x	0.43
Tyrosine	1.49 ± 0.05	2.22	1.78	2.12	1.44	2.27	NA*	0.047	1.58 + 0.01x	0.33
Sum amino acids (PPV)	0.96 ± 0.03	1.23	1.02	1.16	0.92	1.02	NA*	NS	-	-

* AAPV calculations not reliable (i.e. rendering very large or negative values) due to low and variable concentrations of AAs in the samples of these groups.

^a^ n = 4,

^b^ mean value of two crates (n = 2);

BA0 to BA100: insect larvae grown on media with 0% to 100% ground brown algae (0% = pure plant based medium); x = percent inclusion of brown algae in feeding media (0–100).

The total amount of Non-Protein Nitrogen Compounds in the fly larvae was ~150 μmole g^-1^ dried larvae (~6–7% of the total amino acids). Using 100% seaweed in the feeding media (BA100) increased the concentration of total Non-Protein Nitrogen Compounds in the BSF larvae compared to when they were grown on BA0 (Table 7). The concentration of free histidine increased with ~50% from BA0 to BA100 (from ~8–12 mg per kg, dry matter) and methionine doubled from 0.5 to 1 mg per kg. The most abundant Non-Protein Nitrogen Compounds were glutamine, alanine and ethanolamine.

**Table 7:** Most abundant Non-Protein Nitrogen Compounds (μmole g^-1^, dry matter) of black soldier fly larvae grown on increasing inclusions of brown algae in feeding media.

	BA0^a^	BA50^b^	BA100^a^	P value	Y =	R^2^
**Essential amino acids**						
Histidine	7.9 ± 0.2	7.7	12.5 ± 0.6	<0.0001	7.92–0.06x + 0.001x^2^	0.97
Isoleucine	1.3 ± 0.0	2.0	1.8 ± 0.2	0.0004	1.28 + 0.02x -0.0002x^2^	0.87
Leucine	1.9 ± 0.1	2.6	2.4 ± 0.2	0.002	1.88 + 0.02x – 0.0002x^2^	0.80
Lysine	1.5 ± 0.0	3.0	2.6 ± 0.1	<0.0001	1.52 + 0.05x – 0.0004x^2^	0.96
Methionine	0.5 ± 0.0	0.8	1.0 ± 0.1	<0.0001	0.47 + 0.009x – 0.0004x^2^	0.97
Phenylalanine	0.7 ± 0.0	1.1	0.8 ± 0.1	NS	-	-
Threonine	4.1 ± 0.2	3.0	2.5 ± 0.2	<0.0001	4.01–0.02x	0.92
Tryptophan	3.3 ± 0.1	0.2	0.3 ± 0.1	<0.0001	3.25–0.09x + 0.0006x^2^	0.99
Valine	3.4 ± 0.2	3.2	4.1 ± 0.2	0.005	3.37–0.01x + 0.0002x^2^	0.72
**Non-essential amino acids**						
Ammonium chloride	8.6 ± 1.0	8.8	13.7 ± 1.7	0.002	8.12 + 0.05x	0.68
Arginine	9.3 ± 0.8	7.2	8.7 ± 0.3	NS	-	-
Ethanolamine	17.8 ± 0.6	21.3	41.1 ± 3.3	<0.0001	17.8–0.09x + 0.003x^2^	0.96
Alanine	15.9 ± 1.1	13.7	16.0 ± 0.9	NS	-	-
Asparagine	4.9 ± 0.3	3.1	3.3 ± 0.2	<0.0001	4.90–0.06x + 0.0004x^2^	0.91
Glutamic acid	6.7 ± 0.3	7.7	7.9 ± 0.6	0.005	6.76 + 0.01x	0.61
Glutamine	26.8 ± 0.8	13.1	20.5 ± 2.3	NS	-	-
Glycine	6.6 ± 0.2	4.1	6.8 ± 0.5	NS	-	-
Proline	14.9 ± 1.4	14.1	7.3 ± 0.7	0.0001	14.9 + 0.05x – 0.001x^2^	0.90
Serine	2.6 ± 0.2	4.4	3.3 ± 0.3	NS	-	-
Phosphoethanolamine	6.9 ± 1.1	6.6	11.1 ± 2.7	0.02	6.43 + 0.04x	0.43
Sum Non-Protein Nitrogen Compounds (μmole g^-1^)*	151 ± 4	134	181 ± 5	<0.0001	151–1.0x + 0.013x^2^	0.95
Sum Non-Protein Nitrogen Compounds (mg g^-1^)*	18.1 ± 0.6	15.2	19.9 ± 0.3	<0.0001	18.1–0.13x + 0.002x^2^	0.94

^a^ n = 4,

^b^ mean value of two crates (n = 2);

BA0: insect larvae grown on plant-based control feeding medium; BA50 and BA100: insect larvae grown on feeding media where 50% and 100% of the control media was replaced with ground brown algae; x = percent inclusion of brown algae in feeding media (0–100);

* includes all measured Non-Protein Nitrogen Compounds (available as supporting information, S2 Table).

#### Fatty acid concentrations of larvae

**Fig 2:**
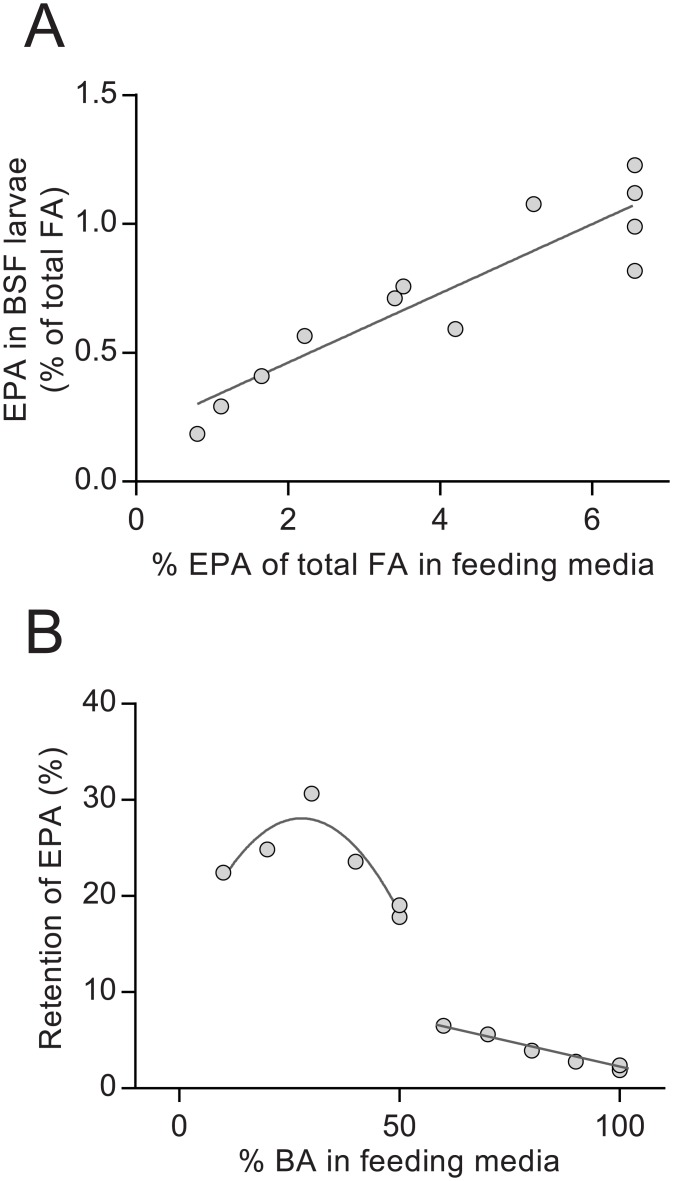
EPA concentration and retention. The concentration of eicosapentaenoic acid (EPA, as % of lipid) in the black soldier fly larvae increased with increased concentrations of EPA in the feeding media, but the retention of EPA decreased dramatically with inclusion of more than 50% brown algae (BA) in the feeding media. Figures show (A) EPA concentrations (% of total fatty acids) of feeding media and black soldier fly larvae with 0–100% of a plant-based feeding media replaced with ground brown algae; and (B) percent of EPA in feeding medium retained by the insect larvae. The BA0 group is not included in the graphs or the calculations, as EPA levels in the larvae as well as in the feeding media was below limit of quantification (0.01 g kg^-1^).

The concentration of total fatty acids in larvae grown on the BA0 medium was more than five times higher than in the larvae grown on BA100 (33.2 and 5.7 g per 100 g dried larvae, respectively) (Table 8). The fatty acid composition of all larvae was dominated by saturated fatty acids, making up between 67.9 and 52.5% of total fatty acids (BA0 and BA100, respectively) (Table 8). The main saturated fatty acid was 12:0, which made up 40.6 and 23.9% of total fatty acids in larvae grown on BA0 and BA100, respectively. By using seaweed in the feeding media, the concentrations of PUFA in the insect larvae increased. The percentage of 18:1n-9 was doubled from 8.8 to 17.9% of total fatty acids when changing composition of feeding media from containing no seaweed (BA0) to containing only seaweed (BA100). Also the longer-chained 20:5n-3 (EPA) and 20:4n-6 (ARA) increased, from not being detected in the BA0 larvae to 1% EPA and 2% ARA of total fatty acids in the BA100 larvae. EPA concentrations in the larvae increased linearly with the EPA concentrations in the media (Fig 2a). The retention of EPA was ~20% of added EPA in the BA0-BA50 groups and dropped to ~2.5–5% with more than 50% seaweed inclusion (Fig 2b). Total amount of EPA was highest in the BA50 larvae, due to their higher lipid levels compared to the BA100 larvae (0.14 and 0.06 g EPA 100/g dry larvae, respectively).

**Table 8:** Fatty acid composition (area %) and total fatty acids (FA, % of dry weight) of black soldier fly larvae grown on increasing inclusions of brown algae in feeding media.

	24H	BA0^a^	BA10	BA20	BA30	BA40	BA50^b^	BA60	BA70	BA80	BA90	BA100^a^	FO [25, 29]	VO [25, 29]	BSF [12]	P value	Y =	R^2^
**12:0**	40.2 ± 1.4	40.6 ± 2.2	38.7	37.9	33.4	33.9	30.5	23.0	21.6	25.5	21.4	23.9 ± 2.9	-	-	42.3	<0.0001	41.33–0.31x + 0.001x^2^	0.88
**14:0**	8.7 ± 0.1	8.5 ± 0.1	8.8	9.4	9.0	8.2	7.3	5.8	5.8	6.6	6.2	6.7 ± 0.5	6.3	0.1	6.9	<0.0001	8.76–0.03x	0.65
**16:0**	14.8 ± 0.3	14.8 ± 0.7	15.6	16.6	17.8	17.2	18.1	19.0	19.1	17.6	17.7	16.6 ± 0.1	11.0	4.7	11.1	<0.0001	14.71 + 0.12x - 0.001x^2^	0.87
**18:0**	2.5 ± 0.2	2.5 ± 0.3	2.8	3.2	3.4	3.4	3.3	4.1	4.3	4.2	4.4	4.1 ± 0.1	0.9	1.6	1.3	<0.0001	2.45 + 0.03x - 0.0001x^2^	0.88
**Total SFA**	67.6 ± 1.2	67.9 ± 1.3	67.2	68.3	64.8	63.9	60.6	53.3	52.3	55.3	50.9	52.5 ± 3.5	18.9	7.5		<0.0001	68.53–0.17x	0.86
**16:1n-7**	1.9 ± 0.2	2.0 ± 0.0	1.8	1.8	1.8	1.7	1.6	1.7	2.0	2.0	2.3	2.5 ± 0.1	8.8	-	-	<0.0001	1.98–0.02x + 0.0002x^2^	0.94
**18:1n-9**	8.9 ± 0.2	8.8 ± 0.3	9.0	9.1	9.7	9.8	10.6	13.3	15.0	14.7	17.9	17.9 ± 1.1	6.7	56.7	12.3	<0.0001	8.67 + 0.01x + 0.0009x^2^	0.88
**Total MUFA**	12.2 ± 0.2	12.2 ± 0.6	13.2	14.5	13.8	12.9	13.3	16.6	18.6	18.4	21.9	22.2 ± 1.3	56.5	62.2	12.3	<0.0001	12.50–0.007x + 0.001x^2^	0.92
**18:2n-6 LA**	18.3 ± 0.7	17.9 ± 0.7	17.5	15.1	18.4	19.0	21.4	24.3	23.5	21.1	20.5	18.6 ± 0.9	1.3	19.5	3.6	NS	-	-
**18:3n-3 ALA**	1.4 ± 0.1	1.4 ± 0.0	1.4	1.2	1.5	1.7	2.1	2.1	1.8	1.7	1.8	1.6 ± 0.1	0.7	9.4	0.7	0.0003	1.30 + 1.92x - 1.55x^2^	0.60
**20:4n-6 ARA**	<LOQ	<LOQ	0.2	0.4	0.5	0.7	1.0	1.2	1.3	1.0	2.1	2.1 ± 0.4	0.3	-	0.2	<0.0001	-0.04 + 0.02x	0.93
**20:5n-3 EPA**	<LOQ	<LOQ	0.1	0.2	0.3	0.4	0.6	0.8	0.7	0.6	1.1	1.0± 0.2	7.5	-	1.7	<0.0001	0.002 + 0.01x	0.93
**22:6n-3 DHA**	<LOQ	<LOQ	<LOQ	<LOQ	<LOQ	<LOQ	<LOQ	<LOQ	<LOQ	<LOQ	<LOQ	<LOQ	5.7	-	0.6			
**Total n-3**	1.4 ± 0.1	1.4 ± 0.0	1.4	1.4	1.9	2.4	2.7	3.0	2.6	2.4	3.2	3.0 ± 0.4	19.8	9.4	3.1	<0.0001	1.45 + 0.02x	0.81
**Total n-6**	18.3 ± 0.7	18.0 ± 0.7	17.7	15.4	19.0	19.9	22.5	25.8	24.9	22.4	23.0	21.1 ± 1.4	1.3	19.6	3.6	0.001	16.98 + 0.16x - 0.001x^2^	0.55
**n-3/n-6**	0.08 ± 0	0.08 ± 0.0	0.08	0.09	0.10	0.12	0.1 2	0.12	0.11	0.11	0.14	0.14 ± 0.0	15.2	0.5	0.9	<0.0001	0.08 + 0.0006x	0.85
**Total PUFA**	19.7 ± 0.8	19.4 ± 0.7	19.2	16.9	20.9	22.2	25.3	28.8	27.7	24.8	26.4	24.2 ± 1.7	21.1	29.0	6.7	0.0002	18.32 + 0.19x - 0.001x^2^	0.63
**Total FA (%)**	32.4 ± 0.7	33.1 ± 0.8	32.9	24.9	26.9	24.3	24.5	13.6	10.5	10.8	6.0	5.7 ± 0.5	-	-	30.4	<0.0001	34.0–0.29x	0.95

^a^ n = 4,

^b^ mean value of two crates (n = 2);

BA0 to BA100: insect larvae grown on media with 0% to 100% ground brown algae (0% = pure plant based medium); 24H: insect larvae grown on medium containing 100% brown algae only last 24 h before harvest; LA: linoleic acid; ALA: alpha-linolenic acid; EPA: eicosapentaenoic acid; DHA: docosahexaenoic acid; SFA: saturated fatty acids; MUFA: monounsaturated fatty acids; PUFA: polyunsaturated fatty acids; FA: fatty acid; LOQ: limit of quantification (0.01 g kg^-1^ (wet weight) or 0.1 area %), NS: not significant; x = percent inclusion of brown algae in feeding media (0–100).

A fatty acid productive value (FAPV) above 1 indicates a net production of a fatty acid during the trial, while a value below 1 indicates a net consumption of a fatty acid. The FAPV for total fatty acids was three times higher in the BA0 larvae than in the BA100, at 1.5 and 0.5, respectively (Table 9). The FAPV for the total saturated fatty acids, were high and maintained >1 for all groups, and reaching values up to 4 in the BA0 and BA10 groups. Monounsaturated fatty acids also had values above 1, indicating a net production of these fatty acids by the larvae, but quickly fell below 1 as the seaweed inclusion increased to more than 50% of feeding media. The PUFAs had FAPV <1, in all groups.

**Table 9:** Fatty acid productive value (FAPV) of black soldier larvae grown on increasing inclusions of brown algae in feeding media.

	BA0^a^	BA10	BA20	BA30	BA40	BA50^b^	BA60	BA70	BA80	BA90	BA100^b^	P value	Y =	R^2^
**Sum SFA**	4.2 ± 0.1	4.0	3.3	3.7	3.6	2.9	1.3	1.1	2.5	1.2	NA**	<0.0001	4.28–0.03x	0.79
**Sum MUFA**	1.6 ± 0.1	1.5	1.3	1.3	1.4	1.0	0.7	0.4	1.4	0.4	0.1 ± 0.0	<0.0001	1.62–0.01x	0.74
**Sum n-3**	0.4 ± 0.0	0.4	0.3	0.5	0.8	0.8	0.3	0.2	0.3	0.1	0.1 ± 0.0	0.006	3.83 + 10.0x - 1.35x^2^	0.48
**Sum n-6**	0.5 ± 0.0	0.6	0.3	0.5	0.5	0.5	0.3	0.3	0.5	0.3	0.5 ± 0.3	NS	-	-
**Sum PUFA**	0.5 ± 0.0	0.6	0.3	0.5	0.6	0.5	0.3	0.3	0.5	0.3	0.3 ± 0.2	0.020	0.53–0.002x	0.27
**Sum FA**	1.6 ± 0.1	1.5	1.2	1.5	1.5	1.2	0.6	0.5	1.1	0.5	0.5 ± 0.3	<0.0001	4.28–0.03x	0.74

^a^ n = 4,

^b^ mean value of two crates (n = 2);

BA0 to BA100: insect larvae grown on media with 0% to 100% ground brown algae (0% = pure plant based medium); SFA: saturated fatty acids, MUFA: monounsaturated fatty acids, PUFA: polyunsaturated fatty acids, FA: fatty acid.

** too low overall concentrations of this fatty acid in samples of this group led to many FAPVs being negative;

x = percent inclusion of brown algae in feeding media (0–100).

#### Vitamin E composition of larvae

In all groups, the main vitamin E species was α-tocopherol, making up ~50% of total vitamin E (Table 10). The tocopherols made up the main bulk of the vitamin E, being 68%, 90% and 96% of total vitamin E analysed in the BA0, BA50 and BA100 groups, respectively. The remaining vitamin E species were tocotrienols. The concentration of α-tocopherol increased 4x when 50% seaweed was added to the feeding medium (BA50), but did not increase further when 100% seaweed was added (BA100). The addition of 50% and 100% seaweed to the feeding media also led to large increases in γ-tocopherol (>20x increase in BA100) and δ-tocopherol (>300x increase in BA100) in the larvae.

**Table 10:** Vitamin E and sterol composition (mg kg^-1^, dry matter) of black soldier fly larvae grown on increasing inclusions of brown algae in feeding media.

	BA0^a^	BA50^b^	BA100^a^	P value	Y =	R^2^
**Vitamin E**						
α - tocopherol	27.1 ± 1	96.1	102.6 ± 11.8	<0.0001	27.1 + 2.0x - 0.01x^2^	0.96
β - tocopherol	7.8 ± 0.5	10.1	10.4 ± 0.4	<0.0001	7.8 + 0.06x + 0.00004x^2^	0.90
γ - tocopherol	1.2 ± 0.1	20.2	32.3 ± 2.7	<0.0001	1.2 + 0.4x + 0.001x^2^	0.99
δ- tocopherol	0.3 ± 0	43.4	94.2 ± 6.1	<0.0001	-0.4 + 0.9x	0.99
α- tocotrienol	0.5 ± 0	0.9	0.2 ± 0	<0.0001	0.5 + 0.02x + 0.0002x^2^	0.99
β - tocotrienol	7.2 ± 0.8	11.7	4.2 ± 0.3	<0.0001	7.2 + 0.2x + 0.002x^2^	0.96
γ - tocotrienol	9.1 ± 3.4	4.4	4.9 ± 1	0.042	8.6 + 0.04x	0.35
δ- tocotrienol	ND	ND	ND	-	-	-
Total vit. E	53.3 ± 3.9	186.7	248.8 ± 19.8	<0.0001	53.3 + 3.4x - 0.01x^2^	0.98
**Sterols**						
Cholesterol	120	-	143	-	-	-
Desmosterol	ND	-	ND	-	-	-
Fucosterol	48.4	-	1181	-	-	-
Stigmasterol	374	-	238	-	-	-
β-sitosterol	1338	-	824	-	-	-
Total sterols	1881	-	2386	-	-	-

^a^ vitamin E: n = 4, sterols: mean value of two crates (n = 2),

^b^ mean value of two crates (n = 2);

ND: not detected (LOD for δ- tocotrienol: 16 μg kg^-1^; desmosterol: 11.3 mg kg^-1^); BA0: insect larvae grown on plant-based control feeding medium; BA50 and BA100: insect larvae grown on feeding media where 50% and 100% of the control media was replaced with ground brown algae; x = percent inclusion of brown algae in feeding media (0–100).

#### Sterol composition of larvae

The sterol composition of the insects was only analysed in the BA0 and BA100 groups (two crates per group, n = 2). There were large variations between the crates in the concentration of the various sterols, but the sterol profile of the BA0 larvae was more dominated by β-sitosterol and stigmasterol than the BA100 larvae, that was more dominated by fucosterol (Table 10). Cholesterol only made up a small proportion (~6%) of the total sterols.

#### Mineral composition of larvae

The concentrations of most minerals (magnesium, calcium, iodine, iron, sodium and potassium) in the larvae followed the concentrations of the media in a linear fashion (Table 11). Manganese remained stable in the larvae, despite varying concentrations in the media. Phosphorus and copper, however, were 2–3 times lower in the BA100 media than in the BA0 media, but had a higher concentration in the BA100 larvae than in the BA0 larvae. Zinc, which was similar in all the feeding media, doubled in the BA100 larvae compared to the BA0 larvae. Iodine was not detected in the larvae fed on the pure plant control medium (BA0), but increased linearly with seaweed inclusion. The retention of minerals in the larvae (as percent of mineral added via the feeding media) decreased when more seaweed was included in the media for all minerals except for phosphorus (Table 12).

**Table 11:** Mineral composition (Ca, Fe, K, Mg, Mn, Na, P: g kg^-1^, dry matter; I, Cu, Zn, Se: mg kg^-1^, dry matter) of black soldier fly larvae grown on increasing inclusions of brown algae in feeding media.

	BA0^a^	BA10	BA20	BA30	BA40	BA50^b^	BA60	BA70	BA80	BA90	BA100^a^	FM[25]	SP[25]	BSF[28]	P value	Y =	R^2^
**Ca**	8.4 ± 0.4	12.0	15.0	17.0	23.0	20.0	24.0	27.0	25.0	25.0	30.0 ± 3.4	22.0	1.8	5.4–61.6	<0.0001	8.7 + 0.3x - 0.001x^2^	0.93
**Fe**	0.21 ± 0.02	0.31	0.36	0.39	0.32	0.30	0.35	0.30	0.34	0.38	0.35 0.01	0.11	0.15	0.10–0.63	<0.0001	0.2 + 0.009x + .0001 x^2^ -0.000001x^3^	0.73
**K**	10.2 ± 0.2	13.0	15.0	18.0	18.0	18.5	20.0	20.0	20.0	21.0	21.3 ± 0.5	10.8	0.8	10.4–18.8	<0.0001	10 + 0.34x – 0.004x^2^ + 0.0002x^3^	0.98
**Mg**	2.1 ± 0.1	2.5	2.7	3.1	3.0	3.3	4.1	4.4	5.1	5.8	6.2 ± 0.2	1.4	0.4	3.5–5.6	<0.0001	2.2 + 0.0013x - 0.0003x^2^	0.98
**Mn**	0.19 ± 0.01	0.18	0.17	0.15	0.17	0.14	0.15	0.19	0.18	0.17	0.17 ± 0.01	0.005	0.39	0.20–0.73	NS	-	-
**Na**	1.0 ± 0.1	1.3	2.5	3.1	3.1	4.7	5.6	5.3	7.2	12	12.3 ± 1.1	5.9	10.0	0.9–2.5	<0.0001	1.1 + 0.017x + 0.009x^2^	0.95
**P**	6.8 ± 0.1	7.4	6.9	7.6	7.3	7.5	9.2	10.0	11.0	11.0	11.3 ± 0.3	16.7	8.0	8.7–13.2	<0.0001	6.8 + 0.01x + 0.0003x^2^	0.92
**Cu**	7.9 ± 0.2	7.5	7.4	7.6	7.2	7.4	8.7	9.1	9.5	10.0	9.9 ± 0.3	5.6	16.0	10.9–34.3	<0.0001	7.9 – 0.08x + 0.002 x^2^ – 0.00001x^3^	0.92
**I**	<LOQ	17	40	58	68	94.5	130	110	140	210	260 ± 27	-	-	-	<0.0001	4.7 + 0.8x + 0.02x^2^	0.96
**Se**	0.1 ± 0.0	0.1	0.1	0.1	0.1	0.1	0.2	0.1	0.2	0.2	0.2 ± 0.0	2.0	0.0	-	NS	-	-
**Zn**	68 ± 1.6	84	86	93	85	75	99	130	150	150	150 ± 5.7	125	40.0	100–270	<0.0001	70 + 0.17x - 0.007x^2^	0.87

^a^ n = 4,

^b^ mean value of two crates (n = 2);

BA0 to BA100: insect larvae grown on media with 0% to 100% ground brown algae (0% = pure plant based medium); LOQ: limit of quantification (for iodine: 2 mg kg^-1^, dry weight); NS: not significant regression; x = percent inclusion of brown algae in feeding media (0–100).

**Table 12:** Mineral retention (% added minerals recuperated in larvae) of black soldier larvae grown on increasing inclusions of brown algae in feeding media.

	BA0^a^	BA10	BA20	BA30	BA40	BA50^b^	BA60	BA70	BA80	BA90	BA100^a^	P value	Y =	R^2^
**Mn**	84.1 ± 5.4	71.9	74.4	55.4	61.6	41.3	47.0	54.0	46.9	53.2	52.2 ± 3.6	<0.0001	84.4–1.1x + 0.008x^2^	0.86
**Fe**	19.3 ± 2.9	21.3	24.7	19.8	14.0	9.6	9.9	8.5	5.8	6.7	4.1 ± 0.3	<0.0001	21.0–0.2x	0.85
**Cu**	26.5 ± 2.4	22.1	22.7	19.2	16.7	13.5	15.3	13.8	11.8	13.0	12 ± 0.5	<0.0001	26.5–0.3x + 0.002x2	0.94
**Zn**	34.4 ± 3.4	35.8	36.8	31.2	24.3	16.1	19.3	20.1	17.8	17.3	16.3 ± 1.3	<0.0001	34.3–0.2x	0.78
**Se**	45.4 ± 7.0	30.9	31.8	24.6	21.7	12.8	15.1	13.1	9.0	8.2	5.8 ± 0.3	<0.0001	44.3–0.8x	0.94
**Ca**	74.5 ± 2.4	54.4	53.6	37.3	38.5	21.1	22.9	19.2	10.5	9.5	9.9 ± 1.0	<0.0001	73.5–1.3x + 0.007x2	0.98
**Na**	4.2 ± 0.4	3.0	4.5	3.4	2.4	2.4	2.2	1.4	1.4	1.9	1.7 ± 0.1	<0.0001	4.0–0.03x	0.79
**K**	21.3 ± 1.5	19.9	23.6	20.7	17.2	12.7	11.7	8.4	6.4	6.2	5.6 ± 0.2	<0.0001	22.4–0.2x	0.92
**Mg**	24.8 ± 2.4	18.3	17.3	13.1	9.6	7.0	7.2	5.1	4.3	4.4	4.0 ± 0.2	<0.0001	24.6–0.5x + 0.003x2	0.98
**P**	31.4 ± 2.5	29.9	30.3	28.8	27.7	23.3	29.9	30.9	31.7	41.8	51.0 ± 3.3	<0.0001	32.6–0.4x + 0.006x2	0.90
**I**	NA	5.1	6.2	4.4	3.3	2.8	2.8	1.7	1.4	1.8	1.8 ± 0.2	<0.0001	7.1–0.1x + 0.0006x2	0.86

^a^ n = 4,

^b^ mean value of two crates (n = 2);

BA0 to BA100: insect larvae grown on media with 0% to 100% ground brown algae (0% = pure plant based medium); NA: not available due to concentrations <LOQ in media and/or larvae; NS: not significant regression; x = percent inclusion of brown algae in feeding media (0–100).

## Discussion

In the present study, BSF were shown to be efficient at using seaweed for growth, in the form of protein and lipids. However, when the percentage of seaweed increased in the feeding media, the total production of larvae per experimental unit decreased up to 25% (BA100) of the control (BA0). Especially seaweed concentrations above 50% dramatically reduced the mass of larvae produced per crate. The size of individual larvae also decreased with increasing seaweed inclusion, with BA100 larvae having only 21% of the average weight of the BA0 larvae at the end of the trial. The media did not have a large effect on the survival of the larvae until seaweed was added at 80% or more. For many of the parameters of growth, there was a decline when seaweed was added up to 50%, and a larger decline was observed with a seaweed inclusion of 60% or more. We hypothesize that the protein content of the seaweed determines the growth of the larvae. Due to the reduced mean size of the larvae and total feed intake per crate at seaweed inclusions over 50%, we suggest that ~7% protein in the media is needed for proper growth and survival of the larvae. The particle size of the brown algae (current trial: 500–2000 μm) might not have been small enough for it to be efficiently used by the insect larvae (preferred: ~150 μm, pers.comm. Daan Biemans). A further reduction of seaweed particle size could improve the growth of the larvae. Additionally, seaweed are known to contain complex polysaccharides, (e.g. Fucose-containing Sulfated Polysaccharides in brown algae [30]) the presence thereof could have affected the performance of the larvae grown on high inclusions of seaweed in the media.

The proximate composition of the BA0 larvae is comparable to previously published data on BSF larvae fed organic material [3,31]. The ash content of the larvae reflected the ash content of the media. Seaweed is high in mineral content [32] that can be transferred to the insect larvae.

The protein content of the larvae is presented as both true protein (sum of anhydrous amino acids) and crude protein (total nitrogen, N-to-protein factor 6.25). However, using crude protein estimated as N x 6.25 would lead to an overestimation of the protein content of the insects. Nitrogen analyses of the larvae revealed quite high concentrations of non-protein nitrogen (19–30% of total nitrogen). We calculated the non-protein N in other studies on BSF larvae [2,28] showing similar values to ours. Chitin, a nitrogen-containing compound found in the cuticle of insects, has been estimated to be ~3–5% of BSF meal [33,34]. These studies used BSF meal containing 5–11% crude protein, and since nitrogen makes up ~7% of the molecular weight of chitin, the contribution of chitin to the non-protein nitrogen would be small (0.4–0.8% of total nitrogen). Other sources of non-protein nitrogen could be ammonia compounds, peptides, nitrates, pigments as well as nucleic acids [35]. Due to the high concentrations of non-protein nitrogen in the BSF larvae, the crude protein calculated using the standard 6.25 N-to-protein factor will be an over-estimation of protein content (crude protein values from the current study were ~40% of dry matter, while true protein gave values at ~30% of dry matter). Commonly reported protein concentrations in BSF are ~40% of dry matter [3,31,34], but this is by using nitrogen-based estimations of crude protein. Earlier publications on BSF have therefore likely over-estimated the protein content of the larvae. A correct estimate of the insect protein content (whether in whole insects or in insect meal) is vital when insects are intended to be used for food or feed purposes.

Insect meal is often suggested as a suitable replacement of fish meal [36]. However, the amino acid composition of the diet has to be balanced when a replacement is done. BSF has earlier been described as limiting in cysteine, methionine and threonine [37]. In feed, methionine is commonly one of the first limiting essential amino acids [25]. BSF in this experiment does not contain high levels of methionine compared to e.g. fish meal [25]. A typical herring fish meal has methionine levels ~ 3% of crude protein, while the BSF in the current trial had methionine levels between 1.3–1.8% of crude protein. Threonine did not seem to be a limiting amino acid in the BSF of this trial. The larvae were able to produce certain amino acids almost lacking in the seaweed-containing media, like tyrosine.

Animals experiencing periods of fast growth usually have large pools of Non-Protein Nitrogen Compounds [38,39]. The total pool of Non-Protein Nitrogen Compounds (50 mM, wet weight) in the BSF larvae of the current trial is comparable to what has been reported in larvae of other diptera species (71 mM in *Calliphora erythrocephala* haemolymph [40] as well as in marine fish larvae (50mM in larvae of *Hippoglossus hippoglossus* 22 days post-hatching [41]. These pools consist of predominantly non-essential amino acids [38], which is confirmed for BSF larvae in the current results. Ethanolamine stood out amongst the Non-Protein Nitrogen Compounds; its concentration was doubled by substituting 100% of the control feeding medium with seaweed, making up ~22% of the total Non-Protein Nitrogen Compounds pool of the BA100 larvae. Ethanolamine is a compound forming part of phospatidylethanolamine, a major phospholipid in animal membranes, which also is abundant in BSF larvae (27% of phosholipids, unpublished results from a pilot trial with BSF fed BA0 feeding media, NS Liland).

The largest lipid fraction (53–68% of total lipid) of the insect larvae was saturated lipid, of which around half was the medium-chained fatty acid 12:0 (lauric acid), which is in line with earlier reports [31]. Lauric acid, which is also the main fatty acid in coconut oil, has been shown to be a very efficient energy substrate for both humans and livestock, as it is efficiently absorbed, digested and β-oxidised (as reviewed by [42]). Due to these properties, medium-chain triglycerides (containing fatty acids with 6- to 12-carbon chains) are used in clinics and by athletes as a quick substrate for energy [42,43]. Medium-chain fatty acids have also been shown to possess antibacterial and antiviral properties and have demonstrated positive effects on gut health [44]. Results from an unpublished pilot trial with BSF larvae showed that the total lipid of BSF consisted of ~90% neutral lipids and that the proportion of neutral to polar lipids did not vary much by changing the feeding media (unpublished results, NS Liland). By growing the larvae on seaweed, the fat was also enriched with EPA and ARA, both important for a normal development of animals [45]. The lipids from the BSF larvae are therefore likely to be very good source for energy in domestic animals.

Based on the FAPV values, it appears that PUFAs were oxidised for energy and that excess energy was stored mainly as saturated- and monounsaturated fatty acids. In a BSF trial where cow manure was replaced with up to 50% fish offal [12], the larvae contained up to 2.3% EPA and DHA of total fatty acids (0.7 g per 100 g insect, dw). In the same study, similar concentrations of EPA and DHA (3.1% of lipids, 0.6 g per 100 g insect, dw) were reached by giving the larvae fish offal only the last 24 hours before harvesting. EPA was present in the larvae grown on seaweed in the current study, but the concentrations were generally low (maximum 1% of total fatty acids, 0.14 g per 100 g insect, dw). Changing to a feeding medium with only seaweed (BA100) for the last 24 hours before harvesting did not have any effect on the fatty acid composition of the larvae.

Of the average 1.2 g EPA added to each BA100 crate of larvae (mean value of four crates), only a mean of 0.03 g were found in the larvae from one crate at the end of the growth period (~2% of added EPA). In the BA50 group, a total of 2.2 g EPA was added per crate (mean value of two crates) during the growth period and 0.4 g was recuperated in each crate (~18% of added EPA). The BA50 group had the highest amount of EPA (0.15 g EPA per 100 g dry whole insect) and the highest recuperation of the same fatty acid. The BA60-BA100 larvae had a dramatically lower growth and lower total lipid levels compared to BA50, most likely leading to more of the PUFA from the media being used for maintenance of basic physiological functions. Although the BA100 group produced less lipids, the lipid they did produce contained a higher percentage of EPA, thus having a more “marine” profile than, for example, the BA50 group.

The mineral composition of the BA0 larvae was within the range of earlier published values of BSF [28]. Elements like sodium and potassium that were present in substantial amounts in the feeding media containing seaweeds, accumulated in the larvae in up to 12 times higher concentrations for sodium. High sodium values might also have affected the growth of the larvae, since it is not known what the tolerance for sodium is for this species of insect larvae. The BA0 larvae contained potassium concentrations comparable to levels found in fishmeal and iodine concentrations many times higher than that reported in other good sources of iodine, like muscle of Atlantic cod (*Gadus morhua*, 12.7 mg kg^-1^ ww [22]; ≤60 mg kg^-1^ BSF larvae, ww). By using seaweed in the feeding media, the larvae were also enriched with calcium, iron, magnesium and potassium, making the insects a good source of minerals.

Seaweed, and especially brown algae like *Ascophyllum* and *Fucus* sp., are known to be a good source of vitamin E [46]. This was reflected in the larvae that had grown on the media with seaweed (*A*. *nodosum*) in the current trial. The larvae growing on BA100 had a more than 6x increase in tocopherols compared to the larvae grown on BA0, making the total vitamin E concentrations higher than typical vitamin E concentrations in dried kelp (150 mg kg^-1^ dried kelp meal, 250 mg kg^-1^ dried BA100 larvae) [25]. All vitamin E species have antioxidant properties and the high concentrations in the larvae fed seaweed might thus protect the nutritional quality of the larvae. Vitamin E can prevent oxidation and peroxidation of PUFAs and protect cell membranes through the same property. The large accumulation of vitamin E in the larvae grown on seaweed therefore improves the nutritional quality of the larvae lipids and the storage quality of the products. The concentrations of sterols in the larvae were generally high, contributing almost purely with plant sterols, which in the whole larvae were comparable to concentrations found in plant oils [47]. Results from the current trial indicate that the larvae do not have a significant production of sterols, but that they merely accumulate sterols from their feeding media.

## Conclusions

From the present study it can be concluded that BSF larvae can be successfully grown on media containing up to 50% *A*. *nodosum*. Replacing a standard medium with *A*. *nodosum* beyond this level led to significant reductions in growth and survival as well as a poorer nutrient utilization. All larvae grown on seaweed were enriched in EPA, iodine and vitamin E, improving the nutrient composition of the larvae. For the development of mass production of BSF larvae using seaweed a finer grinding (<150 μm) of the seaweed biomass is recommended before testing.

## Supporting information

S1 Table The proposed regression design at 11 concentration levels and different replication per level used in this study compared against the ideal 6 concentration levels in triplicate as described elsewhere (Araujo (2008)). (TIF)

S2 Table Non-Protein Nitrogen Compounds (μmole g^-1^, dry matter) of black soldier fly larvae grown on increasing inclusions of brown algae in growth media. ^a^ n = 4, ^b^ mean value of two crates (n = 2); BA0: insect larvae grown on plant-based control growth medium; BA50 and BA100: insect larvae grown on growth media where 50% and 100% of the control media was replaced with ground brown algae.
(PDF)
