# Supplementary figures and images for: Modulation of nutrient composition of black soldier fly (Hermetia illucens) larvae by feeding seaweed-enriched media
Source: PLoS One. 2017 Aug 24;12(8):e0183188. doi: 10.1371/journal.pone.0183188 (PMC5570497; doi:10.1371/journal.pone.0183188)

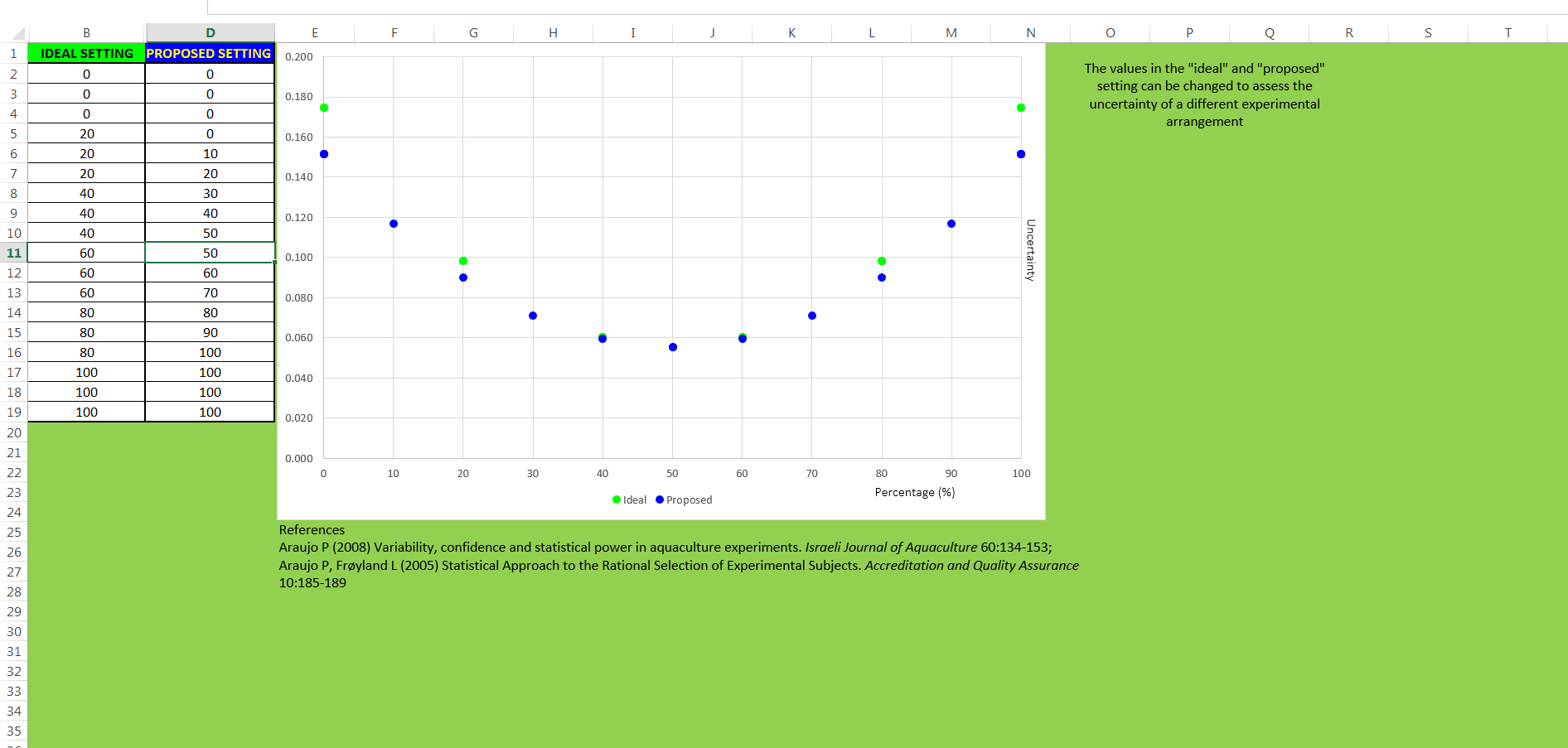

Supplement: S1 Table — (TIF) [file pone.0183188.s001.tif]
