# Supplementary material for: Modulation of nutrient composition of black soldier fly (Hermetia illucens) larvae by feeding seaweed-enriched media
Source: PLoS One. 2017 Aug 24;12(8):e0183188. doi: 10.1371/journal.pone.0183188 (PMC5570497; doi:10.1371/journal.pone.0183188)
Supplement: S2 Table — a n = 4, b mean value of two crates (n = 2); BA0: insect larvae grown on plant-based control growth medium; BA50 and BA100: insect larvae grown on growth media where 50% and 100% of the control media was replaced with ground brown algae. (PDF) [file pone.0183188.s002.pdf]

	BA0[a]	BA50[b]	BA100[a]
**O-Phospho-L-serine**	0.2 ± 0.0	0.2	0.3 ± 0.0
**Taurine**	0.1 ± 0.0	0.4	1.2 ± 0.0
**O-Phosphoethanolamine**	6.9 ± 0.5	6.6	11.1 ± 1.4
**Urea**	0.0 ± 0.0	0.3	1 ± 0.1
**L-Aspartic acid**	0.4 ± 0.0	1.5	1.8 ± 0.1
**L-Threonine**	4.1 ± 0.1	3.0	2.5 ± 0.1
**L-Serine**	2.6 ± 0.1	4.4	3.3 ± 0.1
**L-Aspargine**	4.9 ± 0.1	3.1	3.3 ± 0.1
**L-Glutamic acid**	6.7 ± 0.2	7.7	7.9 ± 0.3
**L-Glutamine**	26.8 ± 0.4	13.1	20.5 ± 1.2
**L-Sarcosine**	0.0 ± 0.0	0.0	2.8 ± 0.2
**L-Proline**	14.9 ± 0.7	14.1	7.3 ± 0.4
**L-glycine**	6.6 ± 0.1	4.1	6.8 ± 0.2
**L-Alanine**	15.9 ± 0.6	13.7	16 ± 0.5
**L-Citrulline**	1.2 ± 0.1	0.3	0.1 ± 0.0
**L-alfa-Amino-n-butyric Acid**	0.0 ± 0.0	0.0	0.1 ± 0.0
**L-Valine**	3.4 ± 0.1	3.2	4.1 ± 0.1
**L-Cystine**	0.0 ± 0.0	0.0	0.0 ± 0.0
**L-Methionine**	0.5 ± 0.0	0.8	1.0 ± 0.0
**Cystathionin1**	0.7 ± 0.1	0.9	3.3 ± 0.3
**L-Isoleucine**	1.3 ± 0.0	2.0	1.8 ± 0.1
**L-Leucine**	1.9 ± 0.0	2.6	2.4 ± 0.1
**L-Tyrosine**	0.3 ± 0.2	0.1	0.1 ± 0.0
**B-Alanine**	0.2 ± 0.0	0.2	0.3 ± 0.0
**L-Phenylalanine**	0.7 ± 0.0	1.1	0.8 ± 0.1
**Gamma-Amino-n-butyric Acid**	1.0 ± 0.1	0.8	0.8 ± 0.0
**Ethanolamine**	17.8 ± 0.3	21.3	41.1 ± 1.7
**Ammoniumchloride**	8.6 ± 0.5	8.8	13.7 ± 0.8
**Hydroxylysine**	0.4 ± 0.0	0.1	0.3 ± 0.0
**L-Ornithine**	1.1 ± 0.1	0.7	1.5 ± 0.1
**L-Lysine**	1.5 ± 0.0	3.0	2.6 ± 0.1
**1-Methyl-L-histidine**	0.2 ± 0.0	0.1	0.2 ± 0.0
**L-Histidine**	7.9 ± 0.1	7.7	12.5 ± 0.3
**L-Trypthophan**	3.3 ± 0.0	0.2	0.3 ± 0.0
**Arginine**	9.3 ± 0.4	7.2	8.7 ± 0.1
**Total FAA**	151.2 ± 1.9	133.6	181.4 ± 2.4
